# Soluble Vascular Cell Adhesion Molecule-1 as an Inflammation-Related Biomarker of Coronary Slow Flow

**DOI:** 10.3390/jcm12020543

**Published:** 2023-01-09

**Authors:** Qing Zhu, Cuiting Zhao, Yonghuai Wang, Lixin Mu, Xinxin Li, Yiqiu Qi, Jun Yang, Chunyan Ma

**Affiliations:** 1 Department of Cardiovascular Ultrasound, The First Hospital of China Medical University, Shenyang 110001, China; 2 Clinical Medical Research Center of Imaging in Liaoning Province, Shenyang 110001, China; 3 Computer Science and Engineering, Northeastern University, Shenyang 110819, China; 4 Key Laboratory of Intelligent Computing in Medical Image of Ministry of Education, Northeastern University, Shenyang 110819, China

**Keywords:** coronary slow flow, soluble vascular cell adhesion molecule-1, sVCAM-1, inflammation, biomarkers

## Abstract

Background: Coronary slow flow (CSF) is an angiographic entity characterized by delayed coronary opacification with no evident obstructive lesion in the epicardial coronary artery. Several studies have shown that the occurrence and development of CSF may be closely related to inflammation. Soluble vascular cell adhesion molecule-1 (sVCAM-1) is a biomarker related to inflammation. The aim of this study was to evaluate the correlation between plasma soluble VCAM-1 level and CSF occurrence and thus the predictive value of VCAM-1 for CSF. Methods: Forty-six CSF patients and thirty control subjects were enrolled. Corrected thrombolysis in myocardial infarction frame count (cTFC) was used to diagnose CSF. Functional status and quality of life were determined by the Seattle Angina Questionnaire (SAQ). Echocardiography was used to evaluate the systolic and diastolic function of the left ventricle (LV) and right ventricle (RV). The plasma levels of sVCAM-1, IL-6, and TNF-α were quantified by enzyme-linked immunosorbent assay. Results: Compared with the control group, the physical limitation score by the SAQ, the LV global longitudinal strain (GLS), mitral E, and mitral E/A decreased in patients with CSF, while the plasma IL-6 and TNF-α levels increased. The plasma sVCAM-1 level in the CSF group was significantly higher than that in the control group (186.03 ± 83.21 vs. 82.43 ± 42.12 ng/mL, *p* < 0.001), positively correlated with mean cTFC (r = 0.57, *p* < 0.001), and negatively correlated with the physical limitation score (r = −0.32, *p* = 0.004). Logistic regression analyses confirmed that plasma sVCAM-1 level (OR = 1.07, 95%CI: 1.03–1.11) is an independent predictor of CSF, and the receiver operating characteristic curve analysis showed that plasma sVCAM-1 levels had statistical significance in predicting CSF (area under curve = 0.88, *p* < 0.001). When the sVCAM-1 level was higher than 111.57 ng/mL, the sensitivity for predicting CSF was 87% and the specificity was 73%. Conclusions: Plasma sVCAM-1 level can be used to predict CSF and was associated with the clinical symptoms of patients. It may serve as a potential biomarker for CSF in the future.

## 1. Introduction

Coronary slow flow (CSF) is an angiographic entity characterized by non-obstructive coronary artery lesions but delayed filling at the end of a coronary artery [1]. We have previously confirmed that left ventricular systolic and diastolic function decrease in patients with CSF [2,3,4]. Therefore, CSF constitutes a serious health problem. Accordingly, clinicians must have access to methods for predicting and diagnosing CSF early, so as to provide timely treatment.

However, owing to the unclear etiology and pathogenesis of CSF, it has no uniform treatment method [5,6]. It is currently believed that CSF may be related to factors such as inflammation, decreased vascular endothelial function, and abnormal microvascular reserve function [4,7,8,9,10,11]. Numerous studies have demonstrated that inflammation plays an important role in the occurrence and development of cardiovascular and microvascular diseases [12,13,14]. Nevertheless, the role of inflammation in the occurrence and development of CSF remains unclear.

Vascular cell adhesion molecule-1 (VCAM-1) is an important cell adhesion molecule that is mainly expressed in cytokine-activated vascular endothelial cells. It plays an important role in the adhesion and migration of leukocytes from circulation to the surrounding tissues and participates in the occurrence and development of inflammatory reactions [12]. In recent years, an increasing number of studies have shown that plasma soluble VCAM-1 (sVCAM-1) is related to cardiovascular disease [15]. However, the relationship between plasma sVCAM-1 and CSF requires further study.

With this background, the present study was undertaken to find out (a) the correlation between the plasma soluble VCAM-1 level and occurrence of CSF and (b) the predictive value of VCAM-1 for CSF.

## 2. Patients and Methods

### 2.1. Patients

We conducted a case–control study with patients that received coronary angiography because of typical angina, coronary risk factors, or abnormal electrocardiography changes from the Department of Cardiology of the First Affiliated Hospital of China Medical University from March 2018 to September 2020, and patients with coronary angiography showing all three epicardial coronary arteries at ≤40% (coronary stenosis not exceeding 40%) were included. Corrected thrombolysis in myocardial infarction frame count (cTFC) was used to diagnose CSF, where subjects with at least one main epicardial coronary artery with cTFC > 27 frames were assigned to the CSF group and those with all three epicardial coronary arteries with cTFC ≤ 27 frames were assigned to the control group [16]. The exclusion criteria were as follows: previous history of myocardial infarction; coronary artery spasm or dilation; abnormal cardiac structure (congenital heart disease, valve dysfunction, or cardiomyopathy); cardiac arrhythmia (ventricular preexcitation, atrial fibrillation, atrioventricular conduction abnormalities, bundle branch block, or pacing rhythm); decreased LVEF (LVEF <53% in men or women [17]); uncontrolled hypertension; hyperthyroidism or hypothyroidism; malignant tumor; autoimmune diseases; known inflammatory or immune diseases; local or systemic infections; lung, liver, kidney, and hematological diseases; and positive exercise stress tests (to distinguish CSF from syndrome X). A total of 46 patients with CSF and 30 age- and sex-matched control subjects were enrolled in the study.

This study was performed in accordance with the Declaration of Helsinki and approved by the Ethics Committee of China Medical University. Written informed consents were obtained from all participants.

### 2.2. Coronary Angiography and TFC

The radial artery approach was chosen for coronary angiography, the standard Judkins technique was used for multi-position angiography, and nicorandil, nitroglycerin, and calcium channel blockers were not used during the operation. Coronary blood flow velocity was evaluated by quantitatively measuring the TFC of the three main epicardial coronary arteries, namely the left anterior descending coronary artery (LAD), left circumflex artery (LCX), and right coronary artery (RCA). The acquisition speed of coronary angiography images in this study was 30 frames/s.

TFC calculation: The last frame number minus the first frame number is taken as the exact number for coronary arteries. The first frame is taken as that showing the contrast agent beginning to fill the coronary artery lumen; the last frame is taken as that showing the contrast agent reaching the distal mark of each coronary artery (it does not need to completely fill the distal mark). The distal end is defined as the distal branch of the LAD, the distal branch of the longest segment of the LCX, and the first branch of the posterolateral artery of the RCA. Due to the long LAD, the number of frames obtained is divided by 1.7 to obtain the corrected TFC for the LAD (cLAD). The sum of TFC values for the RCA, LCX, and cLAD obtained is divided by 3 to calculate the mean cTFC for each subject. TFC analyses are performed by two independent cardiologists, and any disagreements are resolved by a third cardiologist [18,19].

### 2.3. Seattle Angina Questionnaire (SAQ)

The SAQ was performed 24 h or less before coronary angiography. The subjects were required to complete the SAQ within 5 min. The questionnaire has five dimensions and 19 questions, including the degree of physical limitation (question 1), angina stability (question 2), angina frequency (questions 3–4), treatment satisfaction (questions 5–8), and quality of life (questions 9–11). Each question is scored item-by-item, and each score is converted into standard points according to the following formula: standard points = (actual score − lowest score in this aspect)/(highest score in this aspect − lowest score in this aspect) × 100. The score range is 0–100, with higher scores indicating fewer symptoms, better functional status, and better quality of life [20].

### 2.4. Echocardiography

Echocardiography was performed within 72 h of coronary angiography using a Vivid E9 ultrasound system (GE Healthcare, Waukesha, WI, USA) equipped with an M5S phased array probe. An EchoPAC ultrasound workstation (GE Healthcare) was used for offline analysis. Image acquisition and data measurements were according to the recommendations from the American Society of Echocardiography [21].

To assess left ventricular (LV) systolic function, LV ejection fraction (LVEF) and global longitudinal strain (GLS) were measured. To assess LV diastolic function, left atrial volume index, mitral E, mitral A, mitral e’, tricuspid regurgitation velocity, calculated mitral E/A, and mitral average E/e’ were measured. To assess right ventricular (RV) systolic function, RV fractional area change, tricuspid annulus plane systolic excursion (TAPSE), and tricuspid annulus systolic velocity were measured. To assess RV diastolic function, tricuspid E, tricuspid A, and tricuspid e’ were measured.

### 2.5. Detection of sVCAM-1, IL-6, and TNF-α

The levels of the plasma inflammatory factors sVCAM-1, IL-6, and TNF-α were measured within 24 h before coronary angiography. Blood samples were obtained from the anterior cubital vein after the patient had fasted for 12 h. The blood sample was immediately centrifuged for 15 min at 3000× *g* and 4 °C to obtain the plasma. Enzyme-linked immunosorbent assays (ELISAs) were performed to determine plasma sVCAM-1, IL-6, and TNF-α levels (Bioss, Beijing, China). All analyses were performed in triplicate.

### 2.6. Statistical Analysis

Continuous data are expressed as mean ± standard deviation, and independent sample *t*-test was used to compare the differences between continuous variables. Categorical variables are expressed in frequency and percentage. In addition, the chi-square test was used to compare the differences between categorical variables. The Shapiro–Wilk test was used to evaluate whether a variable was normally distributed. Pearson correlation analysis was used to evaluate the correlation between continuous variable parameters. Spearman correlation analysis was used to evaluate the correlation between the data parameters of each grade. Linear regression analysis and binary logistic regression analysis were performed to obtain independent variables related to coronary slow flow. Parameters that were statistically different in the two groups were included in univariate linear regression analyses. Parameters with significance in the univariate linear regression analysis were included in the multivariate linear regression analysis. Logistic regression analysis results are expressed by odds ratio (OR) and 95% confidence interval (CI). Receiver operating characteristic (ROC) curves were used to evaluate the distinguishing ability of the selected variables for predicting CSF and to obtain the cut-off values of independent influencing factors.

Statistical analysis was performed using SPSS 23.0 software package (IBM Corp, NY, USA). For all parameters, *p* < 0.05 (two-tailed) was considered statistically significant.

## 3. Results

The demographic, medical history, laboratory value, medication, and angiographic details of the study population are shown in Table 1. There are no significant differences between the two groups in terms of demographic, medical history, laboratory value, and medication. Patients with CSF have significantly higher TFC values for the cLAD, LCX, and RCA and a higher mean cTFC than the control subjects. In addition, one-, two-, and three-vessel involvements were observed in 26%, 50%, and 24% of the CSF patients, respectively.

The SAQ physical limitation score for the CSF group is significantly lower than that for the control group (56.76 ± 21.84 vs. 68.65 ± 16.30, *p* = 0.008), while there are no significant differences in angina stability, angina frequency, treatment satisfaction, and quality of life between the two groups. These findings indicate that the functional status of CSF patients is decreased (Table 2).

Although there are no significant differences in LVEF between the two groups, LV GLS for the CSF group is significantly lower than that for the control (−17.22 ± 2.23 vs. –18.46 ± 2.37, *p* = 0.04). In addition, the mitral E and mitral E/A for the CSF group are lower than those of the control group (59.76 ± 11.22 vs. 67.77 ± 13.36, *p* = 0.01; 0.87 ± 0.31 vs. 1.05 ± 0.32, *p* = 0.03). These findings indicate that left ventricular systolic and diastolic function in CSF patients are reduced (Table 3).

**Figure 1:**
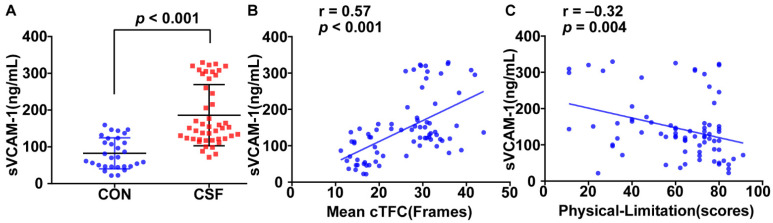
Relationship between sVCAM-1 level and coronary slow flow (CSF). Plasma sVCAM-1 level is significantly higher for patients with CSF than that for the control (**A**) and is positively correlated with mean corrected thrombolysis in myocardial infarction frame count (cTFC) (**B**). Plasma sVCAM-1 level is negatively correlated with SAQ physical limitation score (**C**).

The plasma sVCAM-1 level for the CSF group is significantly higher than that for the control (186.03 ± 83.21 vs. 82.43 ± 42.12 ng/mL, *p* < 0.001, Figure 1A). It is positively correlated with the mean cTFC (r = 0.57, *p* < 0.001, Figure 1B) and negatively correlated with the physical limitation score (r = −0.32, *p* = 0.004, Figure 1C). Moreover, the plasma levels of IL-6 and TNF-α in the CSF group are higher than those in the control group (137.00 ± 57.54 vs. 100.03 ± 24.58 ng/mL, *p* = 0.001; 2.30 ± 0.76 ng/mL vs. 1.93 ± 0.35 ng/mL, *p* = 0.016) and are positively correlated with mean cTFC (*r* = 0.36, *p* = 0.002; *r* = 0.25, *p* = 0.03).

In the correlation analysis, we found that age, sex, body mass index (BMI), systolic blood pressure, smoking history, fasting blood glucose, triglycerides, and total cholesterol were not associated with mean cTFC. Then, we included the parameters that were statistically different between the CSF group and the control group in the univariate linear regression analysis, including physical limitation, mitral E, mitral E/A, TAPSE, LV GLS, IL-6, TNF-α, and sVCAM-1. By univariate analysis, sVCAM-1, IL-6, TNF-α, and LV GLS are associated with mean cTFC. Upon adjusting for baseline covariates, including age, sex, body mass index (BMI), systolic blood pressure, smoking history, fasting blood glucose, triglycerides, and total cholesterol, multivariate linear regression analysis still shows that the association between plasma sVCAM-1 level with mean cTFC is significant (Table 4).

Independent predictors of CSF were investigated using multivariate clinical, echo, and inflammation-related blood models (Table 5). Logistic regression analysis confirmed that the plasma sVCAM-1 level (OR = 1.07, 95%CI: 1.03–1.11, *p* = 0.001) is still an independent predictor of CSF after adjusting for age, sex, BMI, mitral E, and other variables having a *p* < 0.10 by univariate analysis, including physical limitation, mitral E, mitral E/A, LV GLS, TAPSE, IL-6, and TNF-α (Table 5).

**Figure 2:**
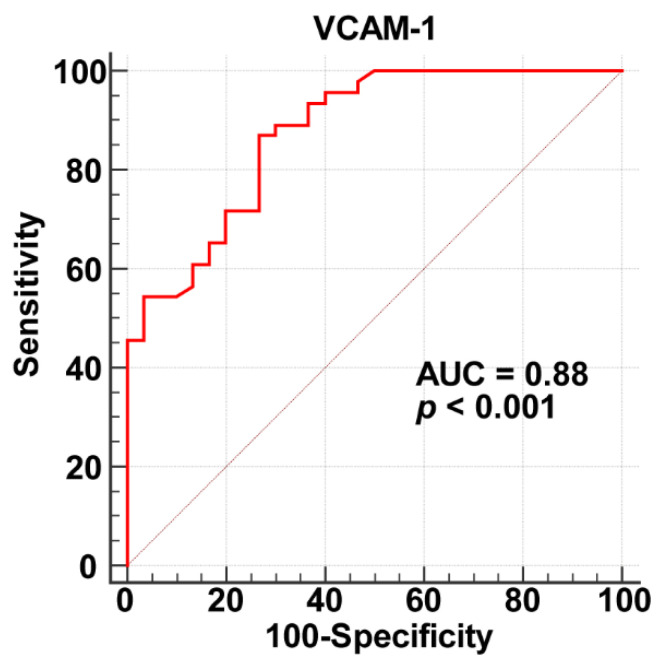
Receiver operating characteristic curve analysis results for plasma sVCAM-1 as a biomarker of CSF.

The ROC curve analysis showed that plasma sVCAM-1 level can predict CSF (area under curve = 0.88, *p* < 0.001, Figure 2) with a cut-off value of 111.57 ng/mL (sensitivity, 87%; specificity, 73%).

## 4. Discussion

We studied the relationship between plasma sVCAM-1 level and CSF, and the main findings are as follows: (1) Plasma sVCAM-1 in the CSF patient group is significantly increased. It is positively correlated with mean cTFC and negatively correlated with the SAQ physical limitation score. (2) Plasma sVCAM-1 level is an independent predictor of CSF. When it is greater than 111.57 ng/mL, the sensitivity for predicting CSF is 87% and the specificity is 73%.

In recent years, an increasing number of studies have focused on patients with coronary angiography showing no obstructive coronary artery disease (CAD) nor structural heart disease that nevertheless suffer from angina-like chest pain. Such conditions may be related to coronary microvascular dysfunction [22]. CSF patients often visit a doctor for angina-like chest pain, whereupon coronary angiography shows that the coronary arteries are normal or close to normal yet filling of their coronary arteries is delayed.

The etiology and pathogenesis of CSF are still unclear, but there is an increasing body of evidence indicating that it is a microvascular disease [22,23]. Inflammation is closely related to cardiac microvascular diseases [24]. For instance, inflammation is implicated in the occurrence and development of diabetic cardiomyopathy [25]. Furthermore, coronary microvascular endothelial inflammation also plays an important role in heart failure with preserved ejection fraction [26]. However, the relationship between inflammation and the occurrence and development of CSF remains unclear.

In assessing the relationship between certain inflammatory biomarkers, oxidative stress parameters, and CSF, Neda et al. confirmed that the pathogenesis of CSF may be closely related to inflammation [8]. Other studies have indicated that the ratio of fibrinogen to albumin and hsCRP to albumin in CSF patients is elevated, as is the level of the inflammation-related plasma biomarker miRNA-155 [11,27]. Our previous studies on the pathogenesis of CSF have also indicated that inflammation plays an important role in the progression of CSF [7]. IL-6 and TNF-α are cytokines that directly promote the responses of blood vessels and inflammatory cells. They are currently recognized as inflammatory biomarkers that are implicated in the inflammatory processes of several diseases. We found that plasma IL-6 and TNF-α levels in CSF patients are increased. However, existing studies have not yet confirmed that plasma IL-6 and TNF-α levels can be used as independent predictors of CSF, so there remains an urgent need to find useful inflammation-related predictors of CSF.

The adhesion of leukocytes to vascular endothelial cells is one of the most important features of the inflammatory process [28]. VCAM-1 is an adhesion molecule of the immunoglobulin superfamily. It is a 90 kDa cell surface glycoprotein expressed in vascular endothelial cells, macrophages, smooth muscle cells, and dendritic cells, among others. VCAM-1 on the surface of endothelial cells binds to α4β1 integrin (also known as VLA-4) molecules distributed on the surface of leukocytes to activate the signaling pathways in endothelial cells, thereby promoting leukocyte adhesion and transendothelial migration [29]. VCAM-1 can detach from the cell surface to form sVCAM-1 in the blood. Thus, increased sVCAM-1 levels are considered as a biomarker of inflammation and endothelial activation [30].

In many human heart diseases, factors such as reactive oxygen species and hemodynamics can enhance the expression of VCAM-1 in the heart, and cell infiltration and inflammation in the heart tissue can lead to continuous heart remodeling, fibrosis, and dysfunction [12]. sVCAM-1 has been shown to be related to the degree of atherosclerosis, and might be used as a biomarker to detect the early stages of atherosclerosis [31], while the onset of atrial fibrillation in the general community has been related to the level of sVCAM-1 [12]. Furthermore, elevated plasma levels of sVCAM-1 have been as predictors of mortality and morbidity in patients with chronic heart failure, endothelial injury in patients with coronary artery disease, and arrhythmias [30]. These studies reveal that sVCAM-1 level is closely related to cardiovascular disease.

Our study has demonstrated that plasma sVCAM-1 levels in CSF patients are significantly elevated, indicating that inflammation plays an important role in the development of CSF. This may be because VCAM-1 on the surface of vascular endothelial cells in CSF patients bound to ligands activates endothelial cells, increases the adhesion of leukocytes and vascular endothelial cells, and enhances inflammatory response. In addition, the level of sVCAM-1 was also positively correlated with mean cTFC, indicating that slower coronary blood flow velocity appeared to be associated with the inflammatory response.

Turhan et al. [32] also found that the patients with CSF had the significantly higher serum ICAM-1, VCAM-1, and E-selectin levels, and the mean TIMI frame count was correlated with plasma sVCAM-1, based on the relatively small sample size (17 patients with CSF and 20 controls). However, the predictive value of sVCAM-1 is unclear, and notably, our study bridged this gap. Our study found that plasma sVCAM-1 levels are an independent predictor of CSF with a sensitivity of 87%, confirming sVCAM-1 as a potential biomarker of CSF. This finding provides a new target and direction for the diagnosis and treatment of CSF.

The SAQ is a widely accepted means by which to assess the quality of life of CAD patients [33]. The physical limitation aspect of the survey quantifies the routine exercise level, specifically captures the limited activity, and reflects the motor function status and quality of life of CAD patients. Perhaps surprisingly, this study revealed that the physical limitation score of CSF patients is significantly reduced, indicating that the patient’s motor function status and quality of life are decreased. Furthermore, plasma sVCAM-1 level was found to be negatively correlated with physical limitation score, indicating that plasma sVCAM1 is not only a plasma biomarker but may also be closely related to a patient’s clinical symptoms.

The main limitation of this research was the small sample size because of the low prevalence and the strict exclusion criteria. Therefore, we designed the case–control study to further control the confounding factors. In addition, a large sample and a prospective and multi-center study are required for further confirmation. Furthermore, because VCAM-1 is synthesized and released by many cell types, such as endothelial cells, macrophages, smooth muscle, and dendritic cells, the determination of the level of plasma sVCAM-1 does not provide any direct information on its origin. Although the findings revealed that VCAM-1 can detach from the cell surface to form sVCAM-1 in the blood, this may also be a limitation in the present study.

## 5. Conclusions

This study revealed that plasma sVCAM-1 is significantly increased in CSF patients. Moreover, sVCAM-1 is an independent predictor of CSF and is negatively correlated with the physical limitation score obtained by SAQ. These indicate that plasma sVCAM-1 may serve as a predictive biomarker for CSF.

## Figures and Tables

**Table 1:** Comparison of baseline clinical characteristics and angiographic findings.

	CSF (*n* = 46)	Controls (*n* = 30)	*p* Value
Demographics			
Age (yrs)	56.11 ± 11.39	56.67 ± 8.30	0.82
Male (*n*(%))	25 (54.3)	12(40.0)	0.22
Body mass index (kg/m^2^)	25.63 ± 5.68	25.90 ± 3.41	0.82
Medical history			
Smoking (*n* (%))	10 (21.7)	6 (20.0)	0.86
Hypertension (*n* (%))	20 (43.5)	9 (30.0)	0.24
Diabetes mellitus (*n* (%))	5 (10.9)	3 (10.0)	0.90
Family history of myocardial infarction	7 (15.2)	7 (23.3)	0.37
Laboratory values			
Triglycerides (mmol/L)	1.62 ± 0.97	1.74 ± 0.75	0.57
Total cholesterol (mmol/L)	4.44 ± 1.00	4.73 ± 1.07	0.25
LDL cholesterol (mmol/L)	2.86 ± 0.82	3.06 ± 0.94	0.33
HDL cholesterol (mmol/L)	1.09 ± 0.26	1.16 ± 0.36	0.31
Fasting blood glucose (mmol/L)	5.58 ± 1.58	5.68 ± 1.72	0.81
Red blood cell count (10^12^/L)	4.61 ± 0.35	4.42 ± 0.37	0.10
Red cell distribution width (%)	13.03 ± 0.34	12.94 ± 0.29	0.24
Platelet count (10^9^/L)	222.45 ± 38.88	220.85 ± 46.87	0.33
Platelet distribution width (%)	11.82 ± 1.37	12.09 ± 1.70	0.48
Medications			
Aspirin (*n* (%))	10 (21.7)	6 (20.0)	0.86
ACEI/ARB (n (%))	20 (43.4)	11 (36.7)	0.55
β-Blockers (*n* (%))	24 (52.2)	14 (46.7)	0.64
Calcium channel blocker (*n* (%))	12 (26.1)	6 (20.0)	0.54
Statin (*n* (%))	34 (73.9)	19 (63.3)	0.33
Nitrates (*n* (%))	33 (71.7)	20 (66.7)	0.64
TFC			
cLAD	30.61 ± 5.97	16.26 ± 4.10	**<0.001**
LCX	40.00 ± 6.97	19.63 ± 3.53	**<0.001**
RCA	25.04 ± 8.24	13.73 ± 5.01	**<0.001**
Mean	31.89 ± 4.46	16.54 ± 2.79	**<0.001**
Vessel involved			
1-vessel (*n* (%))	12 (26.09)		
2-vessel (*n* (%))	23 (50.00)		
3-vessel (*n* (%))	11 (23.91)		

Data are presented as mean ± SD or frequency (percentages). Abbreviations: CSF, coronary slow flow; LDL, low-density lipoprotein; HDL, high-density lipoprotein; ACEI, angiotensin-converting enzyme inhibitor; ARB, angiotensin II receptor blocker; TFC, thrombolysis in myocardial infarction frame count; cLAD, corrected left anterior descending coronary artery; LCX, left circumflex artery; RCA, right coronary artery.

**Table 2:** Comparison of Seattle Angina Questionnaire.

	CSF (*n* = 46)	Controls (*n* = 30)	*p* Value
Physical limitation	56.76 ± 21.84	68.65 ± 16.30	0.008
Angina stability	68.48 ± 33.10	68.52 ± 22.57	0.99
Angina frequency	74.57 ± 21.57	79.09 ± 19.00	0.40
Treatment satisfaction	75.06 ± 17.80	79.96 ± 11.72	0.18
Quality of life	51.45 ± 26.89	51.51 ± 17.36	0.99

Data are presented as mean ± SD. Abbreviations: CSF, coronary slow flow.

**Table 3:** Comparison of left and right ventricular function.

	CSF (*n* = 46)	Controls (*n* = 30)	*p* Value
LV end-diastolic diameter (mm)	47.20 ± 3.86	47.10 ± 3.18	0.92
LV end-systolic diameter (mm)	27.74 ± 3.12	26.90 ± 2.63	0.27
LV end-diastolic volume (mL)	87.62 ± 21.50	84.00 ± 20.51	0.50
LV end-systolic volume (mL)	31.24 ± 7.64	30.75 ± 8.53	0.81
LV ejection fraction (%)	64.16 ± 2.71	63.50 ± 2.95	0.36
LV GLS (%)	−17.22 ± 2.23	−18.46 ± 2.37	**0.04**
LA volume index (mL/m^2^)	27.91 ± 8.51	31.95 ± 10.37	0.16
mitral E (cm/s)	59.76 ± 11.22	67.77 ± 13.36	**0.01**
mitral A (cm/s)	72.51 ± 15.14	67.54 ± 13.11	0.17
mitral E/A	0.87 ± 0.31	1.05 ± 0.32	**0.03**
mitral e’ (cm/s)	7.43 ± 2.19	7.88 ± 1.91	0.41
mitral E/e’	8.47 ± 2.52	8.42 ± 1.86	0.92
TR V (m/s)	2.30 ± 0.46	2.09 ± 0.62	0.24
RV basal diameter (mm)	28.94 ± 4.22	28.49 ± 5.38	0.71
RV fractional area change (%)	46.78 ± 6.56	48.84 ± 6.37	0.18
TAPSE (mm)	25.03 ± 4.32	23.00 ± 4.91	0.09
tricuspid E/A	1.21 ± 0.38	1.36 ± 0.32	0.10
tricuspid S’ (cm/s)	11.72 ± 2.43	11.29 ± 1.68	0.45
tricuspid E/e’	6.47 ± 1.69	5.95 ± 1.56	0.23

Data are presented as mean ± SD. LV, left ventricular; GLS, global longitudinal strain; LA, left atrium; E, early diastolic flow velocity; A, late diastolic flow velocity; e’, early diastolic annular velocity; RV, right ventricular; TAPSE, tricuspid annulus plane systolic excursion; TR V, tricuspid regurgitation velocity; S’, systolic annular velocity.

**Table 4:** Univariable and multivariable liner regression analyses for mean cTFC.

Variables	Univariable		Multivariable			
			Unadjusted		Adjusted	
	*β* [95% CI]	*p* Value	*β* [95% CI]	*p* Value	*β* [95% CI]	*p* Value
Physical Limitation	−0.07 [−0.17–0.02]	0.13				
Mitral E	−0.15 [−0.30–0.01]	0.07				
Mitral E/A	−3.23 [−9.53–3.08]	0.31				
TAPSE	0.44 [0.00–0.88]	0.05				
LV GLS	0.88 [0.02–1.75]	**0.045**	0.40 [−0.35–1.15]	0.29	−0.06 [−0.85–0.74]	0.89
IL-6	0.06 [0.02–0.10]	**0.002**	−0.05 [−0.11–0.02]	0.17	−0.06 [−0.13–0.00]	0.06
TNF-α	3.20 [0.27–6.13]	**0.03**	−2.73 [−6.50–1.04]	0.15	−2.97 [−6.79–0.86]	0.13
sVCAM-1	0.06 [0.04–0.07]	**<0.001**	0.08 [0.05–0.11]	**<0.001**	0.09 [0.06–0.12]	**<0.001**

Model 1—adjusted for age, sex, body mass index, systolic blood pressure, smoking history, fasting blood glucose, triglycerides, and total cholesterol. Abbreviations: cTFC, corrected thrombolysis in myocardial infarction frame count; *β*, regression coefficient; CI, confidence interval; E, early diastolic flow velocity; A, late diastolic flow velocity; TAPSE, tricuspid annulus plane systolic excursion; LV, left ventricular; GLS, global longitudinal strain; IL-6, interleukin-6; TNF-α, tumor necrosis factor-α; sVCAM-1, soluble vascular cell adhesion molecule-1.

**Table 5:** Logistic regression analysis for CSF.

	Model 1		Model 2		Model 3	
	OR [95% CI]	*p* Value	OR [95% CI]	*p* Value	OR [95% CI]	*p* Value
Age	0.99 [0.93–1.04]	0.58	0.93 [0.86–1.01]	0.10	0.94 [0.79–1.11]	0.48
Sex	0.29 [0.09–0.90]	**0.03**	0.34 [0.07–1.78]	0.20	0.84 [0.04–19.64]	0.91
BMI	0.99 [0.89–1.09]	0.79	1.02 [0.90–1.16]	0.79	1.11 [0.93–1.32]	0.26
Physical Limitation	0.95 [0.92–0.98]	**0.003**	0.94 [0.90–0.98]	**0.003**	0.95 [0.88–1.02]	0.16
Mitral E			0.93 [0.86–1.01]	0.09	0.95 [0.83–1.09]	0.46
Mitral E/A			0.36 [0.01–10.35]	0.55	0.13 [0.00–76.76]	0.53
TAPSE			1.16 [0.97–1.40]	0.11	1.37 [0.96–1.96]	0.08
LV GLS			1.17 [0.84–1.61]	0.35	1.24 [0.73–2.10]	0.43
IL-6					0.95 [0.90–1.01]	0.10
TNF-α					0.41 [0.01–12.44]	0.61
sVCAM-1					1.07 [1.03–1.11]	**0.001**

Model 1—Clinical Model: includes age, sex, body mass index, and physical limitation. Model 2—Clinical Model and Echo Model: includes Model 1 plus mitral E, mitral E/A, TAPSE, and LV GLS. Model 3—Clinical Model, Echo Model, and Blood Model: includes Model 2 plus IL-6, TNF-α, and VCAM-1. Abbreviations: CSF, coronary slow flow; OR, odds radio; CI, confidence interval; BMI, body mass index; MV, mitral; E, early diastolic flow velocity; A, late diastolic flow velocity; TAPSE, tricuspid annulus plane systolic excursion; LV, left ventricular; GLS, global longitudinal strain; IL-6, interleukin-6; TNF-α, tumor necrosis factor-α; sVCAM-1, soluble vascular cell adhesion molecule-1.

## Data Availability

The data presented in this study are available on request from the corresponding author. The data are not publicly available due to restrictions eg privacy or ethical.

## References

[1] Tambe A.A., Demany M.A., Zimmerman H.A., Mascarenhas E. (1972). Angina pectoris and slow flow velocity of dye in coronary arteries—A new angiographic finding. Am. Heart J..

[2] Liu S., Wang Y., Li J., Li G., Kong F., Mu L., Jia D., Li Y., Yang J., Ma C. (2021). Incremental Value of Three-dimensional Speckle-tracking Echocardiography for Evaluating Left Ventricular Systolic Function in Patients with Coronary Slow Flow. Curr. Probl. Cardiol..

[3] Wang Y., Ma C., Zhang Y., Guan Z., Liu S., Li Y., Yang J. (2015). Assessment of left and right ventricular diastolic and systolic functions using two-dimensional speckle-tracking echocardiography in patients with coronary slow-flow phenomenon. PLoS ONE.

[4] Zhao C., Zong Z., Zhu Q., Wang Y., Li X., Zhang C., Ma C., Xue Y. (2021). The lncRNA MALAT1 participates in regulating coronary slow flow endothelial dysfunction through the miR-181b-5p-MEF2A-ET-1 axis. Vascul. Pharmacol..

[5] Zhu Q., Wang S., Huang X., Zhao C., Wang Y., Li X., Jia D., Ma C. (2022). Understanding the pathogenesis of coronary slow flow: Recent advances. Trends Cardiovasc. Med..

[6] Chalikias G., Tziakas D. (2021). Slow Coronary Flow: Pathophysiology, Clinical Implications, and Therapeutic Management. Angiology.

[7] Zhu Q., Zhao C., Wang Y., Li X., Xue Y., Ma C. (2021). LncRNA NEAT1 Promote Inflammatory Responses in Coronary Slow Flow Through Regulating miR-148b-3p/ICAM-1 Axis. J. Inflamm. Res..

[8] Roshanravan N., Shabestari A.N., Alamdari N.M., Ostadrahimi A., Separham A., Parvizi R., Jafarabadi M.A., Ghodrat M., Akbarzadeh M., Naemi M. (2021). A novel inflammatory signaling pathway in patients with slow coronary flow: NF-kappaB/IL-1beta/nitric oxide. Cytokine.

[9] Zhao J., Zhang Y., Huang Z., Wu F., Li N., Liang C. (2020). Association between impaired cutaneous microvascular endothelial function and lectin-like oxidized low-density lipoprotein receptor-1 in patients with coronary slow flow. Microvasc. Res..

[10] Niu H., Wei Z., Zhang Y., He J., Jia D. (2018). Atorvastatin improves coronary flow and endothelial function in patients with coronary slow flow. Exp. Ther. Med..

[11] Kayapinar O., Ozde C., Kaya A. (2019). Relationship Between the Reciprocal Change in Inflammation-Related Biomarkers (Fibrinogen-to-Albumin and hsCRP-to-Albumin Ratios) and the Presence and Severity of Coronary Slow Flow. Clin. Appl. Thromb. Hemost..

[12] Willeit K., Pechlaner R., Willeit P., Skroblin P., Paulweber B., Schernthaner C., Toell T., Egger G., Weger S., Oberhollenzer M. (2017). Association Between Vascular Cell Adhesion Molecule 1 and Atrial Fibrillation. JAMA Cardiol..

[13] Moccetti F., Brown E., Xie A., Packwood W., Qi Y., Ruggeri Z., Shentu W., Chen J., Lopez J.A., Lindner J.R. (2018). Myocardial Infarction Produces Sustained Proinflammatory Endothelial Activation in Remote Arteries. J. Am. Coll Cardiol..

[14] Arends C.M., Liman T.G., Strzelecka P.M., Kufner A., Lowe P., Huo S., Stein C.M., Piper S.K., Tilgner M., Sperber P.S. (2022). Associations of clonal haematopoiesis with recurrent vascular events and death in patients with incident ischemic stroke. Blood.

[15] Chyrchel B., Kruszelnicka O., Surdacki A. (2022). Endothelial biomarkers and platelet reactivity on ticagrelor versus clopidogrel in patients after acute coronary syndrome with and without concomitant type 2 diabetes: A preliminary observational study. Cardiovasc. Diabetol..

[16] Gibson C.M., Cannon C.P., Daley W.L., Dodge J.T., Alexander B., Marble S.J., McCabe C.H., Raymond L., Fortin T., Poole W.K. (1996). TIMI frame count: A quantitative method of assessing coronary artery flow. Circulation.

[17] Yao G.H., Deng Y., Liu Y., Xu M.J., Zhang C., Deng Y.B., Ren W.D., Li Z.A., Tang H., Zhang Q.B. (2015). Echocardiographic measurements in normal chinese adults focusing on cardiac chambers and great arteries: A prospective, nationwide, and multicenter study. J. Am. Soc. Echocardiogr..

[18] Wang Y.H., Kang Y.Q., Jin X.Y., Meng P.P., Guan Z.Y., Jia D.L., Gao M.Y., Ma C.Y. (2022). Reference values of the carotid elastic modulus using shear wave elastography and arterial stiffness change in coronary slow flow. Eur. J. Radiol..

[19] Li M., Su H., Jiang M., Zuo Z., Su Z., Hao L., Yang J., Zhang Z., Wang H., Kong X. (2022). Predictive value of thrombolysis in myocardial infarction frame count for coronary microvascular dysfunction evaluated with an angiography-derived index of microcirculatory resistance in patients with coronary slow flow. Quant Imaging Med. Surg..

[20] Spertus J.A., Winder J.A., Dewhurst T.A., Deyo R.A., Prodzinski J., McDonell M., Fihn S.D. (1995). Development and evaluation of the Seattle Angina Questionnaire: A new functional status measure for coronary artery disease. J. Am. Coll Cardiol..

[21] Mitchell C., Rahko P.S., Blauwet L.A., Canaday B., Finstuen J.A., Foster M.C., Horton K., Ogunyankin K.O., Palma R.A., Velazquez E.J. (2019). Guidelines for Performing a Comprehensive Transthoracic Echocardiographic Examination in Adults: Recommendations from the American Society of Echocardiography. J. Am. Soc. Echocardiogr..

[22] Cannon R.O. (2009). Microvascular angina and the continuing dilemma of chest pain with normal coronary angiograms. J. Am. Coll Cardiol..

[23] Kanar H.S., Arsan A., Kup A., Kanar B.G., Tanyildiz B., Akaslan D., Uslu A., Sadic B.O. (2021). Comparison of subfoveal choroidal thickness and retinal nerve fiber layer thickness in patients with coronary slow flow phenomenon and microvascular angina: Optical coherence tomography based study. Photodiagnosis. Photodyn. Ther..

[24] Ma L., Zou R., Shi W., Zhou N., Chen S., Zhou H., Chen X., Wu Y. (2022). SGLT2 inhibitor dapagliflozin reduces endothelial dysfunction and microvascular damage during cardiac ischemia/reperfusion injury through normalizing the XO-SERCA2-CaMKII-coffilin pathways. Theranostics.

[25] Kozakova M., Morizzo C., Goncalves I., Natali A., Nilsson J., Palombo C. (2019). Cardiovascular organ damage in type 2 diabetes mellitus: The role of lipids and inflammation. Cardiovasc. Diabetol..

[26] Paulus W.J., Tschope C. (2013). A novel paradigm for heart failure with preserved ejection fraction: Comorbidities drive myocardial dysfunction and remodeling through coronary microvascular endothelial inflammation. J. Am. Coll Cardiol..

[27] Su Q., Yang H., Li L. (2018). Circulating miRNA-155 as a Potential Biomarker for Coronary Slow Flow. Dis. Markers.

[28] Akbar N., Braithwaite A.T., Corr E.M., Koelwyn G.J., van Solingen C., Cochain C., Saliba A.E., Corbin A., Pezzolla D., Moller Jorgensen M. (2022). Rapid neutrophil mobilisation by VCAM-1+ endothelial extracellular vesicles. Cardiovasc Res..

[29] Kong D.H., Kim Y.K., Kim M.R., Jang J.H., Lee S. (2018). Emerging Roles of Vascular Cell Adhesion Molecule-1 (VCAM-1) in Immunological Disorders and Cancer. Int. J. Mol. Sci..

[30] Troncoso M.F., Ortiz-Quintero J., Garrido-Moreno V., Sanhueza-Olivares F., Guerrero-Moncayo A., Chiong M., Castro P.F., Garcia L., Gabrielli L., Corbalan R. (2021). VCAM-1 as a predictor biomarker in cardiovascular disease. Biochim. Biophys. Acta Mol. Basis Dis..

[31] Llibre J.M., Lopez Cortes L.F., Aylott A., Wynne B., Matthews J., Van Solingen-Ristea R., Vandermeulen K., van Wyk J., Kahl L.P. (2022). Brief Report: Evaluation of Inflammation and Atherogenesis Biomarkers Through 148 Weeks Postswitch to Dolutegravir and Rilpivirine in SWORD-1/SWORD-2. J. Acquir. Immune. Defic. Syndr..

[32] Turhan H., Saydam G.S., Erbay A.R., Ayaz S., Yasar A.S., Aksoy Y., Basar N., Yetkin E. (2006). Increased plasma soluble adhesion molecules; ICAM-1, VCAM-1, and E-selectin levels in patients with slow coronary flow. Int. J. Cardiol..

[33] Spertus J.A., Arnold S.V. (2018). The Evolution of Patient-Reported Outcomes in Clinical Trials and Management of Patients With Coronary Artery Disease: 20 Years With the Seattle Angina Questionnaire. JAMA Cardiol..

